# Modification of Antibiotic Activity by Fixed Oil of the *Artocarpus heterophyllus* Almond against Standard and Multidrug-Resistant Bacteria Strains

**DOI:** 10.3390/biology11121835

**Published:** 2022-12-16

**Authors:** Cícera Janayne Ferreira Dias, António Raposo, Cícera Dayane Thais de Sousa, José Bezerra de Araújo-Neto, Saulo Relison Tintino, Cícera Datiane de Morais Oliveira-Tintino, Isaac Moura Araújo, Henrique Douglas Melo Coutinho, Mayra Garcia Maia Costa, Cleidiane Gomes Lima, Mairlane Silva de Alencar, Conrado Carrascosa, Ariana Saraiva, Erlânio Oliveira de Sousa

**Affiliations:** 1 Laboratory Analysis Physical Chemistry of Food, Faculty of Technology Cariri, Juazeiro do Norte 63041-190, Brazil; 2 CBIOS (Research Center for Biosciences and Health Technologies), Universidade Lusófona de Humanidades e Tecnologias, Campo Grande 376, 1749-024 Lisboa, Portugal; 3 Department of Biological Chemistry, Laboratory of Microbiology and Molecular Biology, Program of Post-Graduation in Molecular Bioprospection, Regional University of Cariri, Crato 63105-000, Brazil; 4 Laboratory of Instrumental Chemistry, Nucleus of Technology and Industrial Quality of Ceará, Fortaleza 60440-552, Brazil; 5 Department of Animal Pathology and Production, Bromatology and Food Technology, Faculty of Veterinary, Universidad de Las Palmas de Gran Canaria, Trasmontaña s/n, 35413 Arucas, Spain

**Keywords:** jackfruit, fatty acid, antibacterial, antibiotic resistance

## Abstract

**Simple Summary:**

The chemical characterization showed a composition of fatty acids that are normally found in other fixed oils that stand out for presenting relevant antibacterial activities alone and/or in association with antibiotics of various classes, such as β-lactams, aminoglycosides, and fluoroquinolones, with lauric acid, myristic acid, oleic acid, and palmitic acid being the main acids in the oil. In this way, further investigations are important to verify if the main compounds present in the oil, in isolation, have antibacterial potential and the modulation capacity verified in the tests carried out with the fixed oil.

**Abstract:**

*Artocarpus heterophyllus* (jackfruit) is an evergreen tree distributed in tropical regions and is among the most studied species of the genus *Artocarpus*. The jackfruit almond has been highlighted in relation to phytochemical studies, biological properties, and application in the development of food products. This study aimed to analyze jackfruit fixed oil regarding chemical components, antibacterial property alone, and in association with antibiotics against standard and MDR bacteria strains. In the analysis of the oil by gas chromatography coupled to a flame ionization detector (GC-FID), a high content of saturated fatty acids (78.51%) was identified in relation to unsaturated fatty acids (17.07%). The main fatty acids identified were lauric acid (43.01%), myristic acid (11.10%), palmitic acid (6.95%), and oleic acid (15.32%). In the antibacterial analysis, broth microdilution assays were used. The oil presented minimum inhibitory concentration (MIC) ≥ 1024 μg/mL in antibacterial analysis for standard and MDR bacterial strains. The oil showed synergistic effects in the association with gentamicin, ofloxacin, and penicillin against MDR strains, with significant reductions in the MIC of antibiotics. The results suggest that the fixed oil of *A. heterophyllus* has fatty acids with the potential to synergistically modify antibiotic activity.

## 1. Introduction

Bacterial resistance to several classes of antibiotics has promoted the search for new compounds and/or associations of compounds with antibacterial activities [1]. In particular, antibiotics of the β-lactam, aminoglycosides, and fluoroquinolones classes are used in several treatments of bacterial infections [2]. Bacterial resistance to antibiotics of these classes has led to a problem in recent years [2,3]. For example, because of resistance, one of the problems with antibiotics of the aminoglycoside class is the toxicity that is linked to high doses or chronic treatment, which causes ototoxicity and/or nephrotoxicity [1].

Bacterial resistance mechanisms act in three main ways: inactivation of antibiotics by hydrolysis or chemical modification; modification of the antibiotic’s specific target involving gene mutation or post-translational process; and reduced intracellular concentrations of the antibiotic as a result of poor penetration or extrusion mechanisms [4].

Several mechanisms are indicated to justify the antibacterial activity of substances from natural products, whether of animal or plant origin. In particular, fatty acids are nonpolar substances from fixed oils that have shown antibacterial activity mainly through an action on the cell membrane, increasing the permeability of antibiotics and interfering with bacterial enzymatic systems integrated into the membrane, such as the energy production or efflux systems [2,5].

In this aspect, studies have indicated the bacterial activity of fixed oils, either in the direct inhibition of bacteria or in association with antibiotics [6,7]. This strategy is known as “herbal shotgun” or “synergistic targeting of multiple effects” and is based on the application of metabolites obtained from plants or animals, due to their structural diversity, in association with antibiotics to affect different bacterial targets and collaborate in a synergistic–agonistic way [8,9].

Fixed oils have shown a strong tendency to change the action of antibiotics against various Gram-positive and Gram-negative strains of standard and MDR bacteria. Oils of pequi (*Caryocar coriaceum*), buriti (*Mauritia flexuosa*), babassu (*Orbignya speciosa*), and mangaba (*Harconia speciosa*) showed antibacterial activity and also synergistic or antagonistic effects in association with aminoglycoside, β-lactam, and fluoroquinolone antibiotics against strains of standard and MDR bacteria, mainly *Escherichia coli*, *Pseudomonas aeruginosa*, and *Staphyloccus aureus*. The action was mainly related to changes in the permeability of the plasmatic membrane by fatty acids [1,3,6,7].

The species *A. heterophyllus* is popularly called jackfruit and stands out in the genus *Artocarpus* in relation to phytochemical studies, biological properties, and application in the development of food products [10,11]. *Artocarpus* species are known for their fruits, which are characterized by having a high content of micro- and macronutrients and bioactive compounds that represent an agro-industrial potential [12].

The consumption of jackfruit is versatile; the pulp can be used in natura; cooked; or as jams, jellies, ice cream, and mousses [12]. Of particular importance, jackfruit almonds are rich in nutrients, such as proteins, carbohydrates, lipids, fibers, and minerals, representing about 15% of the fruit’s weight. It has been used in human food for a long time, being consumed after being roasted in the oven, roasted on a fire, or cooked, as well as in the formulation of various food products such as breads, cookies, cereal bars, kibbehs, dairy drinks, and cakes, among others [12,13].

Review works present several constituents with varied structural patterns that were isolated from almond and other parts of jackfruit, including terpenoids; phytosteroids; prenylated chromones; and a significant number of phenolic compounds, mainly flavonoids. The constituents were analyzed and showed a diversity of biological and pharmacological activities that mainly include antioxidant, anti-inflammatory, cytotoxic, anticancer, anti-HIV, antiproliferative, antidiabetic, and antibacterial effects [5,14].

However, there are no studies dealing with the evaluation of the antibacterial property of *A. heterophyllus* almond fixed oil. Thus, this work becomes relevant, aiming to analyze the chemical composition of the fixed almond oil in terms of fatty acids and verify the antibacterial potential alone or in association with antibiotics against MRSA and other MDR bacterial strains.

## 2. Materials and Methods

### 2.1. Plant Material

Fruits of *A. heterophyllus* (jackfruit) were obtained in an area of Chapada do Araripe, Crato Municipality, Ceará, Brazil. The fruits obtained were selected by separating those that showed mechanical lions and/or by microorganisms. Then, they were subjected to hygienization in a 1% sodium hypochlorite solution for 15 min and then rinsed in running water. The fruits were manually pulped with stainless steel knives, and the obtained almonds were ground in an industrial blender (model LSB-25 SN 010097, Skymsen, Brazil). The almonds were packed in polyethylene plastic packages and subsequently frozen in a freezer (model EFH3, Esmatec, Brazil) at −20 °C.

### 2.2. Extraction of the Fixed Oil

The fixed oil was extracted from 250 g of ground almonds in an industrial blender and subjected to the continuous method in a Soxhlet extractor in triplicate. Hexane was used as a solvent at an average temperature of 60 °C for 3 h. At the end of the process, the solvent was submitted to rotary evaporation (model Q-344B, Quimis, Brazil) under conditions of reduced pressure and controlled temperature of 50 °C.

### 2.3. Determination of Fatty Acids in the Fixed Oil

The fatty acids present in the fixed oil were determined indirectly from the corresponding methyl esters. Methyl esters were obtained by breaking the ester bond of glycerides, fatty acid esters, and glycerol present in the fixed oil. Therefore, the fixed oil was submitted to the process of transesterification reaction. In this process, 0.1 g of the oil was submitted to a saponification reaction with a solution of potassium hydroxide in methanol and left under reflux for 30 min. With proper treatment and pH adjustment, the free fatty acids were then methylated by acid catalysis to produce the respective methyl esters [15].

Methyl esters were analyzed by chromatography using CG Focus, Thermo, provided with an FID in temperature set at 250 °C and fused silica capillary column SP-2560 (100 m × 0.25 mm I.D.; 0.20 µm). The temperature was programmed as follows: 70 to 240 °C at 10 °C/min, totaling the analysis time of 30 min. The temperatures programmed for the injector and detector were 250 °C and 280 °C, respectively.

Nitrogen was the carrier gas used at a flow rate of 1.0 mL/min and split mode (1:10). Injected volume was 1 µL of the solution at a concentration of 1000 g/mL in dichloromethane. Chromatographic standards of fatty acids (C4–C24) and 37 components of the Fame Mix (47885-U Supelco) in a 1:1 ratio in dichloromethane were used. The concentration of the identified fatty acids was calculated from the GC peak area.

### 2.4. Antibacterial Analysis of the Fixed Oil

#### 2.4.1. Preparation of Analysis Substances

For antibacterial analyses, fixed oil and the following antibiotics were used: gentamicin (aminoglycosides class), norfloxacin (fluoroquinolone class), and penicillin (β-lactam class). Antibiotics were obtained from SIGMA (Chemical Co., St. Louis, MO, USA). In preparing the solution for analysis, 10 mg of each substance diluted in 1 mL of dimethyl sulfoxide (DMSO) was used. Then, a dilution in sterile distilled water was performed until we obtained a concentration of 1024 µg/mL.

#### 2.4.2. Bacterial Material

In the antibacterial assays, standard and MDR bacterial strains were used. The standard strains of ATCC (American Type Collection Culture) used were *Escherichia coli* EC-ATCC 25922, *Pseudomonas aeruginosa* PA-ATCC 9027, and *Staphyloccus aureus* SA-ATCC 25923. The MDR strains used were *E. coli* EC-06, *P. aeruginosa* PA-24, and *S. aureus* SA-10, with origin and resistance profiles identified in Table 1. The strains used were obtained from the Laboratory of Microbiology and Molecular Biology of the Regional University of the Cariri. Strains were maintained on blood agar culture medium (Laboratory Difco Ltd., Curitiba, Brazil) and were cultivated at 37 °C for 24 h in heart infusion agar culture medium (HIA, Difco. Laboratorises Ltd.).

#### 2.4.3. Elaboration and Standardization of Bacterial Inoculum

As proposed by Pereira et al. [8], from the cultured bacterial colonies, inoculums were prepared, and the bacteria were added in test tubes with 5 mL of sterile saline solution of 0.9% NaCl. The tubes with the bacterial suspensions were placed in a vortex shaker, and the turbidity was compared to the McFarland scale, which corresponds to 10^5^ CFU (colony-forming units).

#### 2.4.4. Determination of the Minimum Inhibitory Concentration (MIC) Determination

To obtain the MIC, Eppendorf tubes were initially prepared containing 100 µL of the inoculum and 900 µL of the 10% BHI (Brain Heart Infusion) liquid culture medium [8]. The sample prepared in the Eppendorf tubes was distributed (number sense) in 96-well microdilution plates to reach a final inoculum concentration corresponding to 105 CFU/mL. To verify microbial growth, the last well was used as a negative control. In the analysis, a solution prepared at a concentration of 10 mg/mL of fixed oil dissolved in DMSO with a final concentration lower than 10% was used (previous pilot assays performed on our lab demonstrated that DMSO concentrations lower 10% do not affect the final result). Distilled water was used to dilute the solution to a concentration of 1024 mg/mL.

The microdilution plates were prepared in triplicate and then incubated for 24 h in a bacteriological oven at a temperature adjusted to 37 °C. To determine the MIC, 20 µL of sodium resazurin, a blue colorimetric developer, were distributed in each well, waiting for a period of 1 h. After this time, the reading was performed so that the change to the pink color indicated bacterial growth and the conservation of the blue color indicated that growth had occurred.

#### 2.4.5. Ability to Modulate Antibiotic Activity

As proposed by Pereira et al. [8], Eppendorf tubes were prepared adding the following amounts: 1162 µL of 10% BHI culture medium; 150 µL of inoculum of each bacterial strain; and a volume of fixed oil equivalent to the subinhibitory concentration, that is, MIC/8 = 128 µg/mL together with the antibiotics. The negative control was prepared using the following volumes: 1350 µL of 10% BHI culture medium, 150 µL of bacterial inoculum and the antibiotic.

The sample prepared in Eppendorf tubes was distributed into 96-well microdilution plates (number sense) by adding 100 µL to each well. Serial microdilution was then performed by adding 100 µL of the antibiotic to the penultimate well. Microdilution was performed separately for each antibiotic, and the plate concentration ranged from 1024 to 0.5 µg/mL. The analysis was performed in triplicate, and the plates were incubated for 24 h at a temperature adjusted to 37 °C. After this time, the reading was performed using sodium resazurin.

#### 2.4.6. Statistical Analysis

The test results were submitted to statistical analysis using the GraphPad Prism 5.0 statistical program. All tests were organized in triplicate, and the results of geometric means were statistically analyzed by the two-way ANOVA test. Bonferroni’s post hoc test was used and considered significant when *p* < 0.05.

## 3. Results and Discussion

### 3.1. Analysis of Fatty Acid Profile of the Fixed Oil

**Figure 1:**
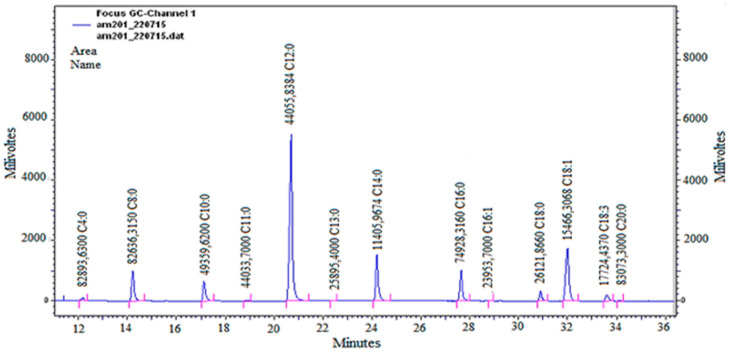
General chromatogram from the analysis of fixed almond oil from *A. heterophyllus* by GC-FID.

The yield obtained for jackfruit fixed oil was 10.20% and it reinforced the previously observed yield of 11.39% [16]. The GC-FID analysis allowed the identification of 95.58% of the fatty acids of the fixed oil chemical composition (Table 2, Figure 1). There was a predominance in the fixed oil of fatty acids of intermediate size (C4 to C20) and a predominance of saturated fatty acids (78.51%) in relation to unsaturated fatty acids (17.07%). Results of the chemical composition of fixed oils usually show fatty acids with intermediate chain (C8 to C24), and some fixed oils also show a predominance of saturated fatty acids [1,7]. Many unsaturated fatty acids identified in fixed oils are also monounsaturated [6].

**Figure 2:**
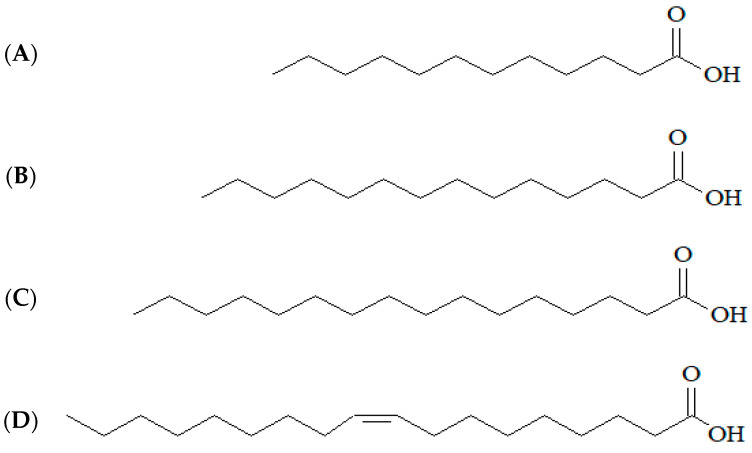
Main constituents identified in the fixed oil of the almond of *A. heterophyllus* and its structural representation: (**A**) lauric acid, (**B**) myristic acid, (**C**) palmitic acid, and (**D**) oleic acid.

The major constituents identified in the oil were lauric acid (43.01%), myristic acid (11.10%), and palmitic acid (6.95%), three saturated fatty acids, and oleic acid (15.32%), a monounsaturated fatty acid (Figure 2). Oleic acid (20.13%), palmitic acid (23.21%), and myristic acid (2.14%) were also major constituents in the composition of the fixed oil of jackfruit [17]. These predominant fatty acids are common and important constituents in terms of biological activities for several other fixed oils [6,8].

### 3.2. Profile of Antibacterial Activity

In the evaluation of the intrinsic antibacterial activity, it was verified that the fixed oil was effective only against the standard strain of *S. aureus* SA–ATCC 25923, with a MIC of 256 µg/mL. For the other standard and MDR strains, the MIC was considered clinically irrelevant (≥1024 µg/mL) (Table 3).

The lowest MIC verified for *S. aureus* corroborates other works that mention the antibacterial property of fixed oils against Gram-positive strains [3,8]. The main constituent of jackfruit oil, lauric acid (Figure 2), has been reported to have bacterial activity, especially against Gram-positive species such as *S. aureus* [18]. On the other hand, Gram-negative bacteria are considered intrinsically more resistant to antibiotics due to the structure of their cell wall, which has an outer membrane with lipopolysaccharides in its constitution, reducing permeability [19].

The study by Mezni et al. [20] corroborates the results obtained, since, by the disc diffusion method, the fixed oil from the fruits of *Pistacia lentiscus* L. inhibited the growth of *S. aureus* and had no effect on *E. coli* and *P. aeruginosa*. Fixed oil from *Nigella sativa* L. seeds, through broth microdilution, was ineffective against Gram-positive and Gram-negative bacteria, with MICs superior to antibiotic controls [21]. Both species have fatty acids in common with jackfruit (e.g., oleic acid), but differences in composition can have different effects [22].

### 3.3. Antibiotic Modifying Activity

**Figure 3:**
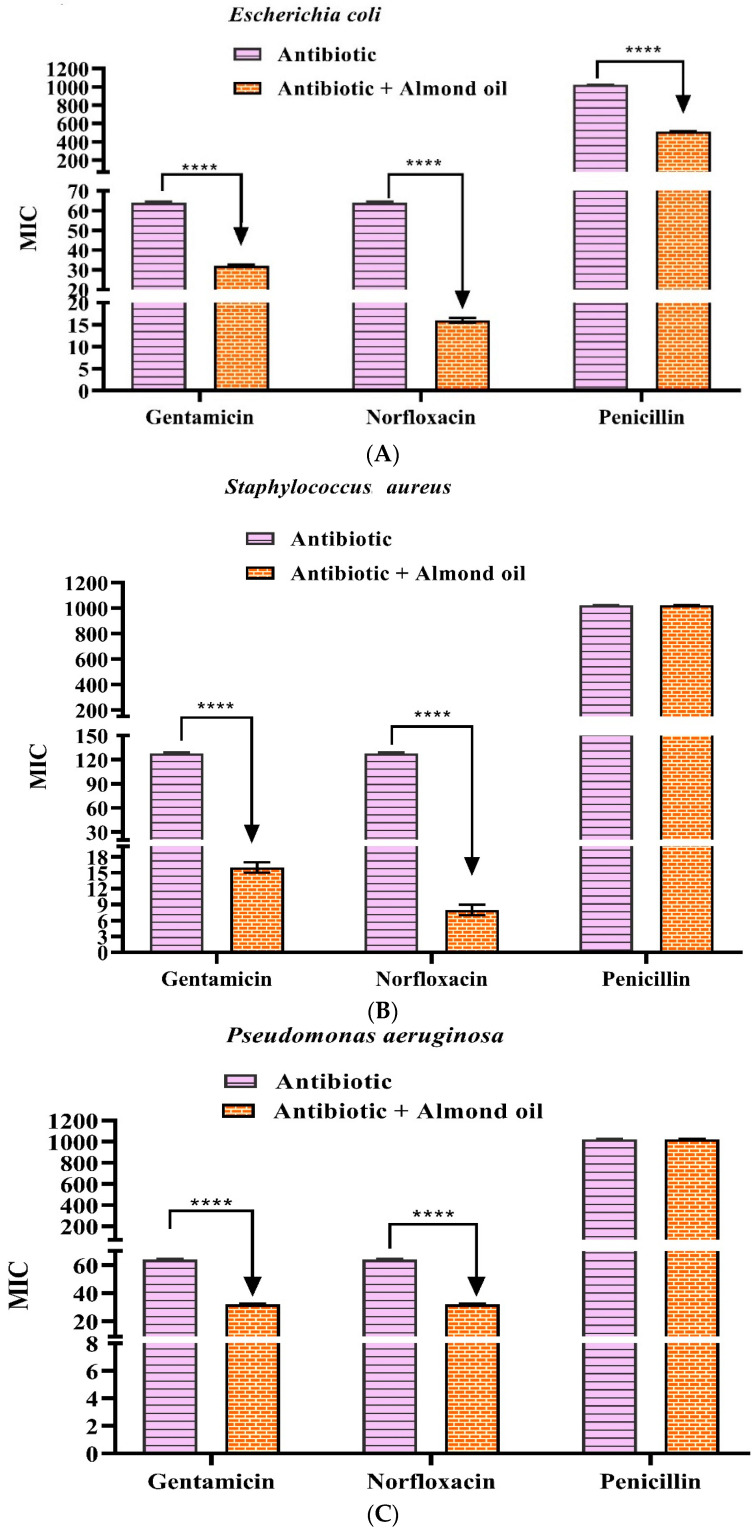
Potentiation of antibiotic action by the fixed oil of the *A*. almond against the analyzed strains: *Escherichia coli* EC–06 (**A**), *Staphyloccus aureus* SA–10 (**B**), and *Pseudomonas aeruginosa* PA–24 (**C**). Values are expressed as geometric mean ± mean standard error (SEM). Two-way ANOVA followed by the Bonferroni test was used. **** *p* < 0.0001, antibiotic alone vs. combined MIC.

The association of jackfruit oil with antibiotics against multidrug-resistant strains resulted, in most cases, in synergism, with a significant reduction in MIC. The main activity was in the association of the oil with norfloxacin against *S. aureus* SA–10, where the MIC was reduced from 128 to 8 µg/mL (16-fold reduction) (Figure 3). Other expressive results were observed in association with gentamicin against *S. aureus* (eightfold reduction) and with norfloxacin against *E. coli* (fourfold reduction). In other cases of synergism, potentiation was 50%. The oil did not change the MIC of penicillin against *P. aeruginosa* PA–24 and *S. aureus* SA–10.

Fixed oils from plant and animal species, such as *Harconia speciosa* [6], *Mauritia flexuosa* [3,8], *Orbignya speciosa* [3,7], *Rhinella jimi* [23], *Gallus gallus* domesticus, and *Meleagris gallopavo* [24], when tested against multidrug-resistant Gram-positive and Gram-negative strains, increased the activity of antibiotics (e.g., aminoglycosides, β-lactams, and fluoroquinolones), but also antagonistic and neutral effects were obtained. The authors related synergism mainly to increased permeability of the plasma membrane caused by fatty acids.

It is not yet clear exactly how fatty acids exert their antibacterial, or drug-enhancing, activities, but studies show that the main target appears to be the bacterial membrane, interrupting several essential metabolite processes that occur there [25]. Therefore, some of the main harmful effects on bacterial cells can be attributed to the detergent properties of fatty acids, which, due to their amphipathic structure, interact with the membrane. As a consequence, they interact with the membrane, creating pores that cause leakage of cytoplasmic content [26]. Therefore, this effect, in addition to causing direct action on the bacteria, can allow the entry of bactericidal or bacteriostatic substances, such as antibiotics. As a consequence, this mechanism may also contribute to the synergistic mechanism observed in the present study [27,28].

Fatty acids have mechanisms of action that may have contributed both to their intrinsic activity and to potentiating the effect of antibiotics, namely, inhibition of Gram-positive cell wall biosynthesis, inhibition of DNA and RNA synthesis, inhibition of protein, plasma membrane disruption, and inhibition of metabolic pathways (e.g., glycolysis) [26].

Furthermore, Dasagrandhi et al. [29] demonstrated that 7,10-epoxyoctadeca-7,9-dienoic acid inhibits the NorA efflux pump of *S. aureus*, an indication of the potential of fatty acids to inhibit antibiotic resistance mechanisms. It is noteworthy that NorA is an efflux pump that acts on norfloxacin resistance [30], and thus the result obtained against *S. aureus* SA–10 (Figure 3) may be related to this inhibitory effect.

Regarding the fact that the oil did not change the MIC of penicillin against *P. aeruginosa* PA–24 and *S. aureus* SA–10, there is still no clear information in the literature that explains these results, but the most parsimonious hypothesis is that the oil was not able to inhibit the resistance mechanisms of these strains to the antibiotic. Resistance to beta-lactams in the species in question may involve modification of the site of action, efflux pumps, reduced permeability, and beta-lactamases enzymes [31,32,33].

There are studies that show that β-lactamase can be inhibited by fatty acids [34]. Therefore, the synergistic effect with beta-lactams may have occurred due to beta-lactamase inhibition. There are also studies that show that fatty acids such as linoleic acid can inhibit efflux pumps [35]. Fatty acids alter membrane permeability, facilitating the entry of antibiotics [28]. It is known that norfloxacin and gentamicin have their action inside the bacterial cell [36]. Therefore, the greater the ease of entering the cell, the better its action will be.

## 4. Conclusions

This was the first study that reported the effect of potentiating the activity of antibiotics in association with *A. heterophyllus* fixed almond oil. The oil showed bacterial activity especially for S. *aureus* and relevant synergistic effects through the reduction of MICs of antibiotics of several classes (β-lactams, aminoglycosides, and fluoroquinolones) against MDR bacterial strains.

The chemical characterization by GC-FID showed that the main acids of the fixed oil composition (lauric acid, myristic acid, oleic acid, and palmitic acid) are normally found in jackfruit fixed oil and in several other oils that have relevant biological properties. New investigations are indicated to verify if the main constituents present in the chemical composition of the fixed oil, alone, have antibacterial action and modulation potential verified in the tests with the fixed oil.

## Figures and Tables

**Table 1:** Bacterial source and profile of the multidrug-resistant strains.

Bacteria	Source	Profile of the Multidrug-Resistant Strains
*E. coli* EC-06	Uroculture	Cephalothin, Cephalexin, Cefadroxil, Ceftriaxone, Cefepime, and Ampicillin + Sulbactam
*P. aeruginosa* PA-24	Nasal	Cefepime, Ceftazidime, Imipenem, Ciprofloxacin, Piperacillin + Tazobactam, Levofloxacin, Meropenem, and Amikacin
*S. aureus* SA-10	Rectal swab	Cephalothin, Cephalexin, Cefadroxil, Oxacillin, Penicillin, Ampicillin, Ampicillin + Sulbactam, Amoxicillin, Moxifloxacin, Ciprofloxacin, Levofloxacin, Erythromycin, Clarithromycin, Azithromycin, and Clindamycin

**Table 2:** Fatty acids identified in the fixed oil of the almond of *A. heterophyllus* after obtaining the corresponding methyl esters using GC-FID.

Nº	Constituents	Nº Carbons	RT (min)	AO (%)
1	Butyric acid	C4:0	12.34	1.74
2	Caprylic acid	C8:0	14.37	8.16
3	Capric acid	C10:0	17.29	4.85
4	Undecanoic acid	C11:0	19.04	0.04
5	Lauric acid	C12:0	20.85	43.01 *
6	Tridecanoic acid	C13:0	22.55	0.03
7	Myristic acid	C14:0	24.37	11.10 *
8	Palmitic acid	C16:0	27.79	6.95*
9	Palmitoleic acid	C16:1^Δ9^	28.99	0.02
10	Stearic acid	C18:0	31.04	2.55
11	Oleic acid	C18:1^Δ9^	32.16	15.32 *
12	Linoleic acid	C18:2^Δ9,12^	33.77	1.73
13	Arachidic acid	C20:0	34.26	0.08
Total saturated				78.51
Total unsaturated				17.07
Total identified				95.58

RT—retention time. * Principal fatty acids. AO: almond oil.

**Table 3:** Results of MIC (μg/mL) in the presence of *A. heterophyllus* fixed almond oil.

Bacterial Strains	MIC (µg/mL)
*Escherichia coli* EC–ATCC 25922	≥1024
*Escherichia coli* EC–06	≥1024
*Pseudomonas aeruginosa* PA–ATCC 9027	≥1024
*Pseudomonas aeruginosa* PA–24	≥1024
*Staphyloccus aureus* SA–ATCC 25923	256
*Staphyloccus aureus* SA–10	≥1024

## Data Availability

Not applicable.
